# On Partial Fracture of the Neck of the Thigh-Bone

**Published:** 1845-01

**Authors:** 


### ON PARTIAL FRACTURE OF THE NECK OF THE THIGH-BONE. By *T. Wilkinson King*. (Guy's Hospital Reports.)

THIS is another contribution to the *vexata quæstio* of the union of the fracture of the cervix femoris in the aged.

" Surgeons are aware that the inferior part or buttress of the cervix, or that part between the head and the trochanter minor, is by far the most solid. All the superincumbent weight is directed on this part, and thus it would seem its comparative hyper-nutrition is excited or induced. It happens, however, that with general senile atrophy and loss of elasticity and agility, this part is particularly prone to give way: the head of the bone sinks on the dense shell, and the inferior fragment is driven up into the cancelli of the head. At the same time, less violence is done to the thin elastic shell of the upper part of the neck of the femur, or that between the head and trochanter major. Possibly it is not a very rare event, that in this way fracture extends through somewhat more than half of the neck."

In the case of a man, æt. 72, the bone was divided, with the exception of one-third of the shell superiorly, and anteriorly. Dr. Colles also describes three cases of partial fracture.

"Considering the fact of partial fracture of the neck as sufficiently established, I wish to connect it with the peculiar mode of nutrition of the head of the femur. The artery which supplies the head of the femur, while it constitutes an epiphysis, is persistent through life. It is a large terminal branch of the internal circumflex, which enters a foramen a little behind and below the highest point of the neck of the femur. After this, it curves over the denser layer of cancelli left by the union of the epiphysis to the shaft, directing its course beyond the insertion of the round ligament, to which I doubt not it furnishes nourishment. Now, it is remarkable that this vessel occupies the situation of the greatest immunity from violence; and that, if only a little periosteum about it escape division when complete fracture occurs, it may be left entire to sustain that which I think could scarcely live without it. This consideration seems corroborated by all the examples I have examined of ligamentous union after fracture at this part. Whether there be a re-union by solid ligament, by a few scattered bands, or by a kind of capsule and cell, (all rare events,) I find the course of this vessel apparently uninterrupted." 349.

In reference to the so-called examples of osseous union, Mr. King observes that the fracture has resulted from atrophy of the part as much as from violence; and, as such atrophy continues also after the injury, it is difficult to imagine how ossific re-union should be going on at the same time. He suggests that, in these cases, the fracture may have been only partial; and considers that the facts he adduces, and the collection of specimens at Guy's, only enforce Sir A. Cooper's well-known opinions upon the subject.

He notices that the branch of the circumflex which supplies the epiphysis of the os humeri, is also so protected that a transverse fracture may occur without destroying the continuity of the arterial channel.

---

